# Low Abundance *Fusobacterium Nucleatum* Supports Early Pregnancy Development – An *In Vitro* Study

**DOI:** 10.3389/fimmu.2021.698045

**Published:** 2021-08-31

**Authors:** Martha Heusler, Rebekka Einenkel, Jens Ehrhardt, Damián Oscar Muzzio, Marek Zygmunt

**Affiliations:** Department of Obstetrics and Gynecology, University of Greifswald, Greifswald, Germany

**Keywords:** microbiome, pregnancy, trophoblast, Fusobacterium nucleatum, HTR8/SVneo, BeWo, JEG-3

## Abstract

Pregnancy success depends greatly on a balanced immune homeostasis. The detection of bacterial components in the upper reproductive tract in non-pregnant and pregnant women raised questions on its possible beneficial role in reproductive health. The local conditions that allow the presence of bacteria to harmonize with the establishment of pregnancy are still unknown. Among the described bacterial species in endometrial and placental samples, *Fusobacterium nucleatum* was found. It has been observed that *F. nucleatum* can induce tumorigenesis in colon carcinoma, a process that shares several features with embryo implantation. We propose that low concentrations of *F. nucleatum* may improve trophoblast function without exerting destructive responses. Inactivated *F. nucleatum* and *E. coli* were incubated with the trophoblastic cell lines HTR8/SVneo, BeWo, and JEG-3. Viability, proliferation, migratory capacity, invasiveness and the secretion of chemokines, other cytokines and matrix metalloproteinases were assessed. The presence of *F. nucleatum* significantly induced HTR8/SVneo invasion, accompanied by the secretion of soluble mediators (CXCL1, IL-6 and IL-8) and metalloproteinases (MMP-2 and MMP-9). However, as concentrations of *F. nucleatum* increased, these did not improve invasiveness, hindered migration, reduced cell viability and induced alterations in the cell cycle. Part of the *F. nucleatum* effects on cytokine release were reverted with the addition of a TLR4 blocking antibody. Other effects correlated with the level of expression of E-cadherin on the different cell lines tested. Low amounts of *F. nucleatum* promote invasion of HTR8/SVneo cells and induce the secretion of important mediators for pregnancy establishment. Some effects were independent of LPS and correlated with the expression of E-cadherin on trophoblasts.

## Introduction

It is estimated that a healthy adult hosts a number of bacteria comparable in magnitude with the number of own human cells. Commonly known, skin, gut and vagina are densely colonized body sites. The colon, the site where most bacteria reside, is estimated to contain around 3.8 × 10^13^ bacteria (1). The gut microbiota has established symbiotic relationships with the host bearing mutualistic advantages for both, bacteria and the host. The human body, thereby, profits from pathogen defense, provision of metabolites and immunological challenges mediating enteric homeostasis. Alterations in its composition, instead, may cause several health problems (2).

The gut microbiome has its origin early in life and its development depends on several factors. The colonization and thus the composition is affected by the mode of birth (3), genetic factors, nutrition and the intake of antibiotics (4–6). In recent years, the hypothesis that the infant gut is colonized *in utero* has gained strength upon reports describing microbial communities in meconium from neonates delivered at full term by C-sections (7–10). The maternal origin of the *in utero* colonization is still under discussion, but maternal gut, uterine and oral microflora have been proposed as source as well (11). This assessment defies the consensus that has been assumed over 100 years that the healthy womb is sterile (12).

During pregnancy, immune homeostasis is crucial for pregnancy maintenance (13). Local and systemic immune adaptations facilitate the implantation and later the accommodation of the growing fetus (14–17). These adaptations include the promotion of uterine vascular remodeling and the induction of immune tolerance (18–24). Both maternal lymphocytes and fetal derived cells including trophoblast establish a complex interaction to balance the inflammatory environment providing protection against pathogens and the necessary cytokine milieu that allows local structural modifications during placentation (17, 25, 26).

Reports supporting the idea of the sterile womb were based on data obtained from culture-based methods. However, considering that only 1 % of the bacteria are cultivable, new methodologic approaches have been applied to revisit the sterile uterine model (27). A number of studies reported the presence of bacteria in healthy uterine cavity, placenta, umbilical cord and amniotic fluid (8, 28–31). Despite that, low bacterial loads were reported which are hardly differentiated from contaminations especially in the placenta (32–35). Furthermore, the mere detection of bacterial genetic material does not imply the presence of living bacteria. In this concern, more research is needed to clarify the impact of bacteria or bacterial products on pregnancy. Nevertheless, it has been speculated that they may play a role in priming fetal immune system or maternal inflammatory processes at the beginning of pregnancy (36, 37).

*F. nucleatum* a non-motile, non-spore-forming, gram-negative bacteria that belongs to the genus *Fusobacterium* of the family *Bacteroidaceae* (38) was found in healthy term placenta (28). It has been described as an opportunistic bacterium of the human oral cavity and one of the most occurrent species causative of periodontitis. Moreover, *F. nucleatum* was found in several organs, and its presence in the colon has been linked to the promotion of carcinogenesis (39, 40).

Many studies have been performed to determine the mechanisms by which *F. nucleatum* is able to modify the tumor niche. The bacterium possesses several virulence factors that suppress immune cells, promote extracellular matrix (ECM) modifications, modify blood vessel formation and induce cell growth (39, 41–48). Thereby, binding of *Fusobacterium* Adhesin A (FadA) to E-cadherin activates β-catenin signalling and promotes direct cancer cell proliferation. The immune suppressive capacity of *F. nucleatum* was demonstrated more than 30 years ago (49). The same authors identified later the *Fusobacterium* immunosuppressive protein (FIP) and its subunit FipA are responsible for the immunosuppressive capacity of *F. nucleatum* (50, 51). Recently, the protein Fap2 was shown to inhibit NK cells *via* TIGIT (T Cell Immunoreceptor With Ig And ITIM Domains), facilitating tumor evasion of the immune system (45). Moreover, *F. nucleatum* can also affect humoral response and monocyte activity (52–54).

Tumor developmental mechanisms show analogies to early pregnancy processes. These include the activation of pathways that promote cell motility. For example, the reduction of the expression of adhesion molecules as E-cadherin facilitates the loss of cell-cell interactions and the epithelial-mesenchymal transition (55, 56). Analogous to trophoblast invasion, tumor growth is also accompanied by modifications of the ECM (57) where matrix metalloproteinases (MMPs) play a fundamental role. It has been observed that *F. nucleatum* promotes tumorigenesis by increasing the release of MMPs. Indeed, *F. nucleatum* stimulates secretion of several MMPs from epithelial cells and macrophages (42, 43, 58) and acquires MMP-9 activity after binding of pro-MMP-9 (41). The FadA target protein, E-cadherin, is also expressed on trophoblasts in a time and location dependent manner during placental development (59–61). Expressed prominently on cytotrophoblasts in anchoring cell columns and villous trophoblasts, its expression is inversely proportional to the cell migratory capacity, being lower in extravillous trophoblasts (EVT). It has been observed that E-cadherin expression also is reduced from first to third trimester of pregnancy. While alterations in the expression of E-cadherin are associated with aberrant placentation (60), the impact of E-cadherin in cancer progression seems to depend on the cancer entity (62).

An infection can affect pregnancy not only by its virulence characteristics, but also by shifting the above mentioned inflammatory equilibrium (63). It has been proposed that placental inflammation is predominantly caused by maternal activation of TLRs (64). As shown in clinical trials, targeting bacterial infection does not warrant prevention of pregnancy complications (65). Hence, understanding immune functions at the fetomaternal interface is highly relevant. Recent studies unveiled the presence of low bacterial abundance in locations previously thought to be sterile [including endometrium, fallopian tubes (66–68) and placenta (28, 29)]. The fact that bacteria or bacterial components may be present at the fetomaternal interface challenges our understanding of local immune homeostasis.

We speculate that the presence of small numbers of *F. nucleatum* in the fetomaternal unit may influence trophoblast invasive capacity, by promoting ECM modifications and a tolerogenic surrounding micro-environment. In this work, we evaluate the effect of non-infective low concentrations of *F. nucleatum* on trophoblast biology.

## Material and Methods

### Cell Lines and Culture

HTR8/SVneo cells (LGC Standards, Wesel, Germany), a human first trimester extravillous trophoblast immortalized cell line, were cultured in RPMI 1640 (PAN-Biotech, Aidenbach, Germany) supplemented with 10% FBS (PAN-Biotech, Aidenbach, Germany) and 1% penicillin/streptomycin (PAN-Biotech, Aidenbach, Germany).

JEG-3 (LGC Standards, Wesel, Germany) and BeWo (LGC Standards, Wesel, Germany), both human chorioncarcinoma cell lines with similar phenotype to cytotrophoblasts, were cultured in DMEM/F12 (Thermo Fisher Scientific, Schwerte, Germany) supplemented with 10% FBS and 1% penicillin/streptomycin. All cells were cultured at 37°C and 5% CO_2_ under humidified conditions.

### Preparation of Inactivated Bacteria for Stimulation

#### F. nucleatum

*F. nucleatum* culture was kindly provided by Elsa Baufeld (Friedrich-Loeffler-Institut, University Medicine Greifswald) after growth on BD Columbia Agar with 5% Sheep Blood (BD, Franklin Lakes, USA) under anaerobic conditions in BD GasPak (EZ pouch system BD, Franklin Lakes, USA). As obligate anaerobes, bacteria were killed by exposure to oxygen for at least 72 h keeping their structure unaltered (69). Inactivated bacteria were scraped off with sterile inoculating loops and washed in phosphate buffered saline (PBS; PAN-Biotech, Aidenbach, Germany). After centrifugation for 30 min at 4°C and 12 000 × *g* supernatant was discarded and the pellet was resuspended in PBS.

For stimulation, inactivated bacteria were used in a serial 10-fold dilution to cover a range of MOI (multiplicity of infection) between 10 and 1 000 times lower than MOI commonly used for *in vitro* infections (45, 70–72).

#### E. coli

*E. coli* was cultured in LB medium (Lennox; Carl Roth, Karlsruhe, Germany) overnight. The suspension was centrifuged for 30 min at 4°C and 12 000 × *g*. The pellet was resuspended in 96% ethanol (Carl Roth, Karlsruhe, Germany) and incubated for 5 min to inactivate the bacteria, keeping their structure unaltered. Afterwards the suspension was washed and resuspended in PBS. As done with *F. nucleatum*, only inactivated bacteria were used in the experiments.

Bacterial concentration was calculated measuring the optical density assessed by the IMPLEN Nanophotometer as performed by Tuttle and colleagues (73).

### Invasion Assay

4 × 10^5^ HTR8/SVneo cells were treated with inactivated *F. nucleatum* (bacteria:cell ratio of 1:100 = 0.01, 1:10 = 0.1, 1:1 = 1, 10:1 = 10) or 10 ng/mL LPS for 6 h. Conditioned media (CM) was collected and spheroids were consequently created in 5% methyl cellulose in U-well plates overnight. Spheroids were embedded in matrigel (10 mg/mL; Corning, New York, USA). After polymerization at 37°C for 2 h the collated CM was added. The growth of cell branching structures (“Sprouting”) was observed and documented at the light microscope (Zeiss, Oberkochen, Germany). The area formed by connected sprout tips was measured at 0 h, 24 h and 48 h and analyzed with ImageJ.

### Cell Migration

Cell migration of HTR-8/SVneo and BeWo was assessed in a scratch assay. 2 × 10^5^ trophoblast cells were cultured in a 24-well plate. Confluent cell monolayer was scratched with a pipette tip. Medium was aspirated and cells were rinsed with warm (37°C) PBS twice. Afterwards control media or stimulation media (positive control EGF (Biomol, Hamburg, Germany) 40 ng/mL; inactivated *F. nucleatum/E. coli* 2 × 10^3^; 2 × 10^4^; 2 × 10^5^; 2 × 10^6^) were added. The cell-free area was measured at 0 h and 12 h (HTR8/SVneo) or 30 h (BeWo) (Zeiss, software: ZEN 2012 SP2) with ImageJ software and MRI Wound Healing Tool macro.

### Cell Viability

Cell viability of HTR8/SVneo, JEG-3 and BeWo was determined after stimulation with inactivated *F. nucleatum* using the CellTiter-Blue^®^ Cell Viability Assay (Promega, Mannheim, Germany). The assay is based on the capacity of living cells to convert resazurin (a redox dye) into resorufin (a fluorescent product). 5 × 10^3^ cells per well were cultured in a 96-well plate. After 30 min incubation, *F. nucleatum* suspensions were added (0; 500; 5 × 10^3^; 5 × 10^4^). After 2, 24 or 48 h incubation 20 µL CellTiter-Blue^®^ was added and incubated for 1 h at 37°C. Fluorescence was measured with BMG FLUOstar OPTIMA Microplate Reader.

### In-Cell Western Assay

E-cadherin expression was determined by In-Cell Western Assay. 2 × 10^4^ cells per well were cultured in a 96-well plate and incubated for 3 h at 37°C to assure adequate attachment. Cells were fixed with 3.7% formaldehyde (Carl Roth, Karlsruhe, Germany) in PBS for 20 min at room temperature. Subsequently, cells were permeabilized by adding cold methanol (Carl Roth, Karlsruhe, Germany) and shaken on ice for 20 min. Cells were then washed with PBS and blocked with Odyssey Blocking Buffer (LI-COR Biotechnology, Bad Homburg, Germany) for 90 min at room temperature. The cells were then incubated with primary antibody (E-Cadherin (24E10) Rabbit mAb, Cell Signaling Technology, Leiden, Netherlands) diluted in Odyssey Blocking Buffer at 4°C overnight. Cells were washed with washing buffer [PBS; 0.1% Tween 20 (Carl Roth, Karlsruhe, Germany)] and incubated with secondary antibody (IRDye^®^ 800CW Goat anti-Rabbit IgG (H + L), LI-COR Biotechnology, Bad Homburg, Germany) and DRAQ5 (Cell Signaling Technology, Leiden, Netherlands), as a normalization control for cell number, diluted in antibody buffer (Odyssey Blocking Buffer; 0.2% Tween 20) for 60 min at room temperature. The cells were washed with washing buffer. The plate was measured with Li-Cor Odyssey Infrared Imager and analysed with Image Studio (LI-COR Biotechnology, Bad Homburg, Germany).

### Apoptosis Rate and Cell Cycle Analysis

Apoptosis rate was determined using the FITC Annexin V Apoptosis Detection Kit II (BD Biosciences, Heidelberg, Germany) according to manufacturer’s instructions. Cell cycle analysis was performed with propidium iodide (PI; Sigma-Aldrich, Schnelldorf, Germany) flow cytometric assay (74). For both experiments cells were cultured in a 48-well plate. After 30 min incubation, inactivated *F. nucleatum* were added (0; 3 × 10^3^; 3 × 10^4^; 3 × 10^5^). After 2, 24 or 48 h incubation the cells were detached and stained. Measurement was done using a BD FACSCanto Flow Cytometer. Data was analysed with FlowJo software.

For cell cycle analysis, cells were stained with 50 μg/ml PI in hypotonic lysis buffer [0.1% Trinatriumcitrat-2-hydrate (Carl Roth, Karlsruhe, Germany); 0.1% Triton-X-100 (Sigma-Aldrich, Schnelldorf, Germany)]. The measurement followed immediately applying a BD FACSCanto Flow Cytometer. The FlowJo cell cycle analysis tool with univariate pragmatic model by Watson (75) was used to differentiate between G0/1; S; G2/M phases.

### Determination of Cytokine- and Matrix Metalloproteinases Secretion

The secretion of cytokines including chemokines (IL-6, IL-8, CXCL1; IL-1β) and matrix metalloproteinases (MMP-2, MMP-9) was determined by ELISA (human IL-6; CXCL8/IL-8; CXCL1/GRO-α; IL-1β; MMP-2; MMP-9 DuoSet ELISA, R&D, Abingdon, United Kingdom). 1 × 10^5^ cells per well were cultured in a 24-well plate and incubated for 30 min at 37°C. The cells were then treated with 0; 10^3^; 10^4^ or 10^5^ inactivated *F. nucleatum* or 10^5^ inactivated *E. coli*. Supernatants were collected after 2 h; 4 h; 8 h; 24 h or 48 h and centrifuged for 10 min at 4°C and 13 000 × *g* to remove dead cells and bacteria and stored at -80°C. The ELISAs were performed according to manufacturer’s protocol. BMG FLUOstar OPTIMA Microplate Reader was used to assess colour changes and calculate the concentrations.

### TLR4 Blocking

5 × 10^4^ HTR8/SVneo cells per well were cultured in a 48-well plate and incubated for 30 min at 37°C. PAb-hTLR4 (TLR4 blocking antibody; InvivoGen,Toulouse, France) was added. After 1 h incubation the cells were stimulated with 5 × 10^4^ inactivated *F. nucleatum.* Supernatants were collected after 48 h and stored at -80°C.

### Multiplex Assay

5 × 10^4^ HTR8/SVneo or 10^5^ BeWo cells per well were cultured in a 48-well plate. After 1 h incubation the cells were stimulated with inactivated 5 × 10^4^ *F. nucleatum*. After 48 h, the supernatant was discarded, and the cells were lysed following the protocol provided by the analyzing kit manufacturer. Proteins (3,7 – 12,2 µg per well as assessed by BCA assay) were analyzed using the NF-κB Signaling 6-plex Magnetic Bead Kit (Merck-Millipore, Massachusetts, USA) and measured in a Bio-Plex 200 System (Bio-Rad Laboratories, Hercules, USA). Data was expressed as fluorescence intensity normalized to the protein amount per well (IF/µg).

### Immunofluorescence

8 × 10^3^ cells per well were seeded in 160 µg/mL collagen G coated µ-Slides (Ibidi, Munich, Germany) and incubated overnight at 37°C in their corresponding media. The following day, TLR4 (PAb-hTLR4 (5 µg/mL), VIPER (5 µM; TLR4 Inhibitor Peptide Set, Novus Biologicals, Wiesbaden Nordenstadt, Germany) and Pitstop 2 (50 µM; Sigma-Aldrich, Schnelldorf, Germany) were added to the corresponding wells 1 h before treatment with inactivated *F. nucleatum* in a 1:1 proportion. After 1 h stimulation, culture media was discarded and cells were fixed with 4 % paraformaldehyde. Immune staining was performed with Phospho-NF-κB p65 (Ser536) (clone 93H1; 1:200) Rabbit mAb or β-Catenin (clone L54E2; 1:200) Mouse mAb (CellSignalling Technology, Frankfurt, Germany) overnight. The staining with secondary antibodies was performed for 2 h at RT in the dark with Goat anti-Mouse IgG (H+L) Highly Cross-Adsorbed Secondary Antibody, Alexa Fluor 594 (ThermoFisher Scientific, Schwerte, Germany) and Goat anti-Rabbit IgG (H+L) Highly Cross-Adsorbed Secondary Antibody, Alexa Fluor 488 (ThermoFisher Scientific, Schwerte, Germany), both in a concentration of 1:500. Slides were stained with 1 µg/mL Hoechst 33258 and analyzed on a Zeiss Axiom microscope at 60×. The exposure time was set constant for each cell line across experiments (green channel: 840 ms; red channel: 400 ms; blue channel: 17 ms). The quantification of fluorescence signal was performed with ZEN 2012 Blue Edition.

### Statistics

Experiments were performed independently in replicates as described in the figure legends. Data were analyzed by GraphPad Prism 5 and 8. Data were assumed normally distributed. For the effect of bacterial treatment on trophoblast biology concerning invasion, migration, viability, apoptosis, cell cycle and cytokine expression Repeated Measures ANOVA with Dunnett’s multiple comparison post test or Šidák’s multiple comparison test was performed. Significant differences were indicated with asterisks *p_adj_ < 0.05; **p_adj_ < 0.01; and ***p_adj_ < 0.001.

## Results

### High Concentrations of Inactivated *F. nucleatum* Reduce Trophoblast Viability

**Figure 1:**
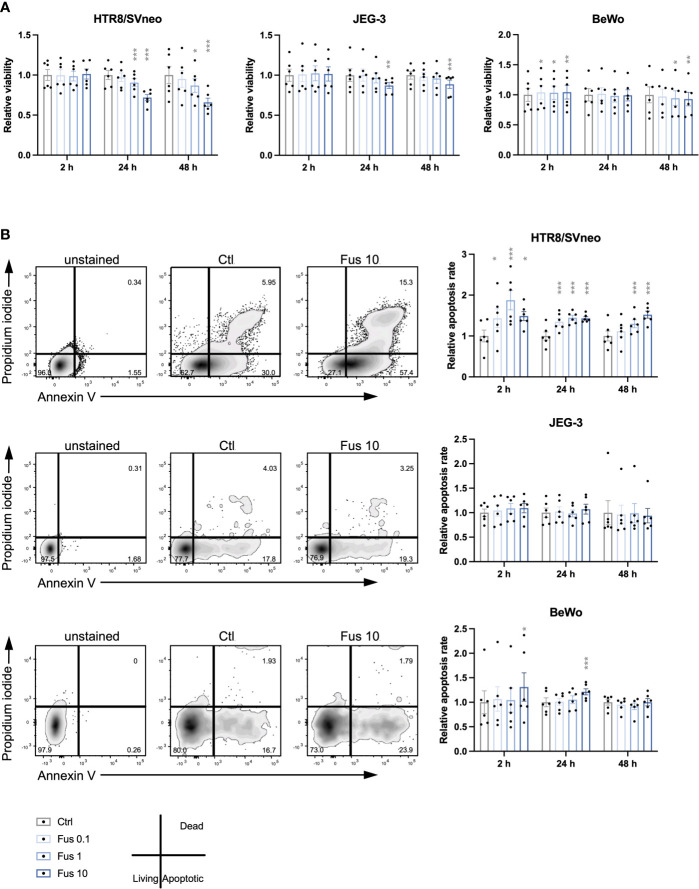
Reduced viability and increased apoptosis rate of HTR8/SVneo cells was seen in response to high concentrations of inactivated *F. nucleatum.* Bar graphs represent viability of trophoblast cell lines after stimulation with *F. nucleatum* normalized to respective controls **(A)**. Representative plots for the analysis of apoptosis rate of HTR8/SVneo, JEG-3 and BeWo cells by flow cytometry (**B** left). Bar graphs show apoptosis rate of trophoblast cell lines after stimulation with *F. nucleatum* normalized to respective controls (**B** right). Normalized data represent the quotient of each value to the mean of untreated controls. Data are presented as mean ± SEM. *p_adj_ < 0.05; **p_adj_ < 0.01; ***p_adj_ < 0.001 as analysed by Repeated Measures ANOVA with Dunnett’s multiple comparison post test, comparing each treatment against the corresponding control. Experiments were performed 6 times in sixtuplicate **(A)** or in triplicates **(B)**. Each point represents the mean value of the replicates for each experiment. Ctl, control; Fus, ratio of *F. nucleatum* to cell number.

During the remodelling of spiral arteries, trophoblast invasion is associated with a constant turnover including cycles of apoptosis and cell growth (76). We assessed cell viability in trophoblasts treated with *F. nucleatum* (**Figure 1A**). No effect on HTR8/SVneo viability was observed at 2 h. Compared to unstimulated control, the viability of HTR8/SVneo cells was significantly reduced after 24 and 48 h after stimulation with *F. nucleatum* concentrations of 1 bacterium per cell and 10 bacteria per cell.

Similar to HTR8/SVneo, JEG-3 viability was significantly reduced after 24 h and 48 h but only by a concentration of 10 bacteria per cell at 24 h and 48 h. In contrast to HTR8/SVneo and JEG-3, BeWo cells showed a different pattern in their viability after treatment with *F. nucleatum*. While all *F. nucleatum* concentrations increased viability after 2 h, concentrations of 1 and 10 bacteria per cell had a negative effect on viability after 48 h.

Overall, we observed that the viability of the cell lines varied in response to treatment with inactivated *F. nucleatum*. High concentrations of inactivated *F. nucleatum* decreased viability of HTR8/SVneo and BeWo cells after 24 and 48 h treatment. In contrast, a short stimulation with bacteria (2 h) enhanced cell viability in BeWo cells.

### Higher *F. nucleatum* Concentrations Increase Apoptosis Rate in HTR8/SVneo and BeWo

Considering the effects of *F. nucleatum* treatment on trophoblast viability, the apoptosis rate was consequently assessed (**Figure 1B**). In HTR8/SVneo, a significant increase of the frequency of apoptotic cells by all *F. nucleatum* concentrations was visible after 2 h and 24 h. After 48 h, the apoptosis rate was increased by *F. nucleatum* concentrations of 1 bacterium per cell and 10 bacteria per cell but not by concentrations of 0.1 bacterium per cell.

In contrast to HTR8/SVneo, the apoptotic rate of both choriocarcinoma cell lines was less affected by inactivated *F. nucleatum*. While apoptosis in JEG-3 cells was not influenced by the treatment, BeWo cells increased apoptosis rate by *F. nucleatum* concentrations of 10 bacteria per cell at 2 h and 24 h.

In terms of induction of apoptosis, HTR8/SVneo cells showed an increased susceptibility to *F. nucleatum* compared to BeWo and especially JEG-3 cells.

### Lower Concentration of *F. nucleatum* Supports Trophoblast Invasion

**Figure 2:**
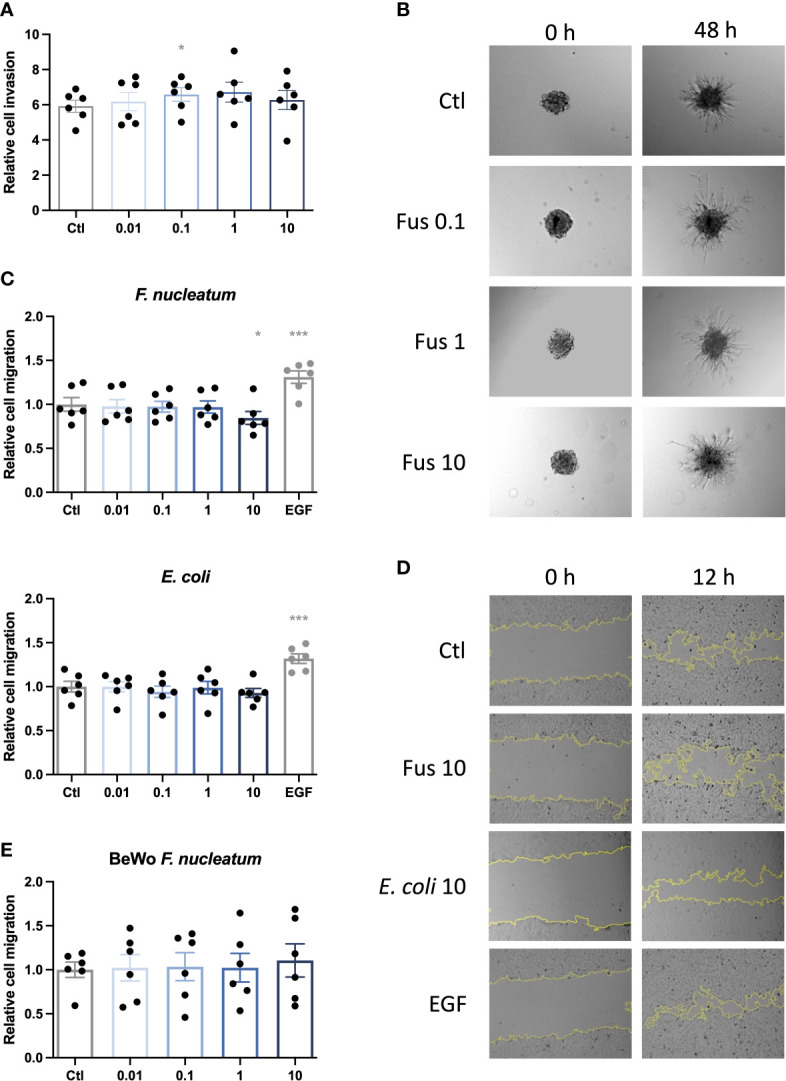
Low concentrations of inactivated *F. nucleatum* promote HTR8/SVneo invasion; high concentration of inactivated *F. nucleatum* impairs migration of HTR8/SVneo cells. HTR8/SVneo cells were stimulated with *F. nucleatum* for 6 h using indicated bacteria:trophoblast ratios. After culture in methyl cellulose-containing medium, spheroids were embedded in matrigel and observed to analyse invasive behaviour **(A, B)**. Bar graph shows relative sprouting expansion after 48 h normalized to spheroid size at 0 h **(A)**. Data are presented as mean ± SEM and were analysed by Repeated Measures ANOVA with Dunnett’s multiple comparison post test, comparing each treatment against the corresponding control. *p_adj_ < 0.05 Representative microscopic images are shown **(B)**. Experiments were performed 6 times. Scratch assay was performed to assess the migratory behaviour of bacteria-treated trophoblasts **(C–E)**. EGF was used as positive control. Inactivated bacteria were added in different ratios (0.01; 0.1; 1; 10 bacteria per trophoblast cell). Bar graphs represent relative area recovered by HTR8/Svneo treated with either *F. nucleatum* (above) or *E*. *coli* (below) after 12 h **(C)** or BeWo treated with *F*. *nucleatum* after 30 h **(E)** normalized to unstimulated control. Data are presented as mean ± SEM. *p_adj_ < 0.05; ***p_adj_ < 0.001 as analysed by Repeated Measures ANOVA with Dunnett’s multiple comparison post test, comparing each treatment against the corresponding control. Experiment was performed 6 times in quadruplicate **(C)** or triplicate **(E)**. Each point represents the mean value of the replicates for each experiment. Representative microphotographs of HTR8/SVneo taken with a 10 × objective taken after 0 and 12 h of the scratch **(D)**. EGF, epidermal growth factor; Ctl, control.

To test our hypothesis that low concentrations of *F. nucleatum* may improve trophoblast invasiveness, an invasion assay using trophoblast spheroids embedded in matrigel was performed (**Figures 2A, B**). After treatment with *F. nucleatum*, the sprouting area formed by connecting sprout tips was assessed after 48 h and normalized to the initial spheroid area at 0 h.

HTR8/SVneo cells tended to increase invasion depth (area formed by the connection of the outer sprout tips) with rising bacterial concentration. Compared to the control, this increase was significant for 0.1, up to 1 bacteria per cell but decreased to control level with higher bacterial concentration (10 bacteria per cell).

### Lower Bacterial Amounts Do Not Affect Trophoblast Migration

Invasion is a complex mechanism of matrix degeneration and cellular motility. In order to determine the mechanisms by which *F. nucleatum* promoted trophoblast invasiveness, we studied effects of bacteria treatment on cell migration. In contrast to the effects observed in invasiveness, no significant effects were observed for the treatment with low concentrations of bacteria up to a ratio of one bacterium per cell. However, treatment with *F. nucleatum* at a ratio of 10 bacteria per cell lead to a significant decrease in the migratory capacity of HTR8/SVneo (**Figures 2C, D**).

*E. coli* treatment did not significantly influence migration of HTR8/SVneo. On BeWo cells, neither *E. coli* (data not shown) nor *F. nucleatum* stimulation had any significant effect on cell migration (**Figure 2C**).

As the re-growth of the scratched area depends not only on cell viability but also proliferation, we moved forward to assess this in trophoblasts treated with *F. nucleatum*.

### *F. nucleatum* Induces Growth Arrest in JEG-3 and BeWo but Turnover in HTR8/SVneo

**Figure 3:**
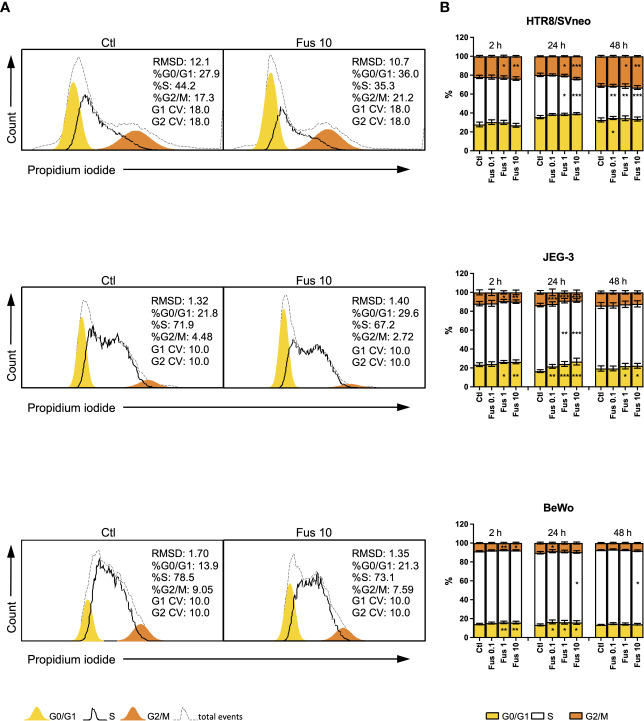
Inactivated *F. nucleatum* increases the frequency of HTR8/SVneo cells in G2/M phase and JEG-3 and BeWo G0/1 phase. HTR8/Svneo, JEG-3 and BeWo were stimulated with different concentrations of *F*. *nucleatum*. Cell cycle analysis was performed after 2, 24 or 48 h. Representative cell cycle analysis of unstimulated control and Fus10 **(A)**. Bar graphs show percentages of cells in the different cell cycle phases **(B)**. Data are presented as mean ± SEM. *p_adj_ < 0.05; **p_adj_ < 0.01; ***p_adj_ < 0.001 as analysed by Repeated Measures ANOVA with Dunnett’s multiple comparison post test, comparing each treatment against the corresponding control. Experiments were performed 6 times in triplicate. Each point represents the mean value of the replicates for each experiment. RMDS, Root Mean Square Deviation; G0/G1 and G2/M main peak modeled as a Gaussian distribution, S calculated; CV, Coefficients of Variation; Ctl, control; Fus, ratio of *F. nucleatum* to cell number.

To test the biological effect of *F. nucleatum* on trophoblast proliferation behaviour, we investigated the cell cycle phases with DNA staining and flow cytometry (**Figure 3**).

In the HTR8/SVneo cell line, *F. nucleatum* induced an increment of the proportion of cells in the G2/M phase at ratios 1 and 10 bacteria per cell. After 24 h, this was accompanied by a decrease of cells in S phase. The effects of 0.1 bacteria per cell were observed only after 48 h. Here, an increment of the of the G0/G1 phase and a decrease of S phase was induced after treatment.

In contrast to HTR8/SVneo cells, JEG-3 cells reacted to the treatment with *F. nucleatum* by through a reduction of the G2/M phase after 2 h (at ratios 1 and 10) and 24 h (all concentrations). These changes were accompanied by an increment of the G0/G1 phase and, after 24 h, a reduction of the S phase. After 48 h, only significant changes in the G0/G1 phase (an increment) could be observed at ratios 1 and 10.

Similar to JEG-3 cells, *F. nucleatum* treatment led to a reduction of the G2/M phase (after 2 h at ratios 1 and 10, after 24 h at a ratio of 0.1) and an accumulation of cells in the G0/G1 phase (after 2 h at ratios 1 and 10, after 24 h for all ratios) in BeWo cells. Ratios of 10 bacteria per cell also reduced the S phase after 24 h and 48 h.

Overall, we observed that *F. nucleatum* treatment led to an increased proportion of cells in G2/M of HTR8/SVneo, but to an accumulation of cells in G0/G1 of JEG-3 and BeWo.

### *F. nucleatum* Treatment Induces Secretion of Pro-Invasive Mediators in HTR8/SVneo but Not in BeWo

Certain pro-inflammatory cytokines, acting paracrinally or autocrinally, promote invasion of trophoblasts. Furthermore, trophoblasts secrete matrix metalloproteinases (MMPs) facilitating the invasion of trophoblasts. We analyzed the effect of *F. nucleatum* treatment on the secretion of pro-inflammatory cytokines and MMPs in trophoblasts cell lines.

**Figure 4:**
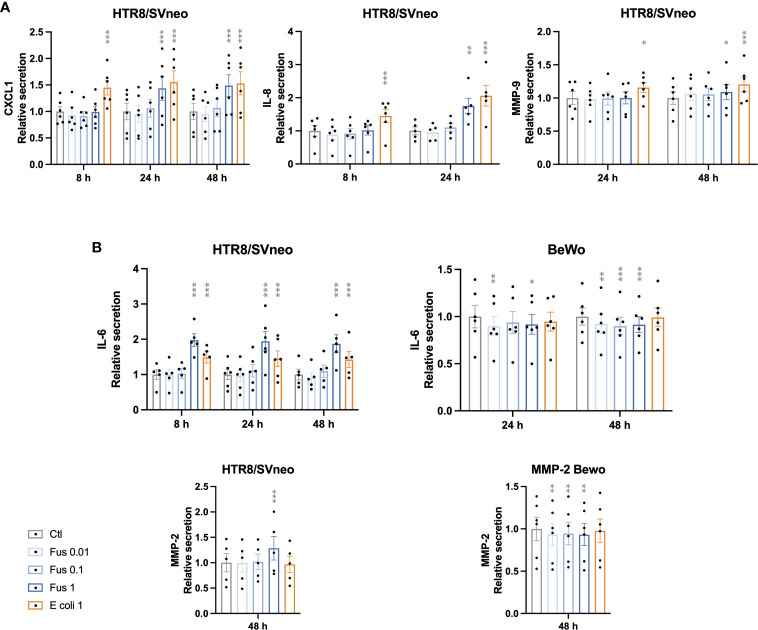
Inactivated *F. nucleatum* and *E. coli* augment secretion of pro-inflammatory cytokines and MMPs by HTR8/SVneo cells. Bar graphs represent secretion of cytokines and matrix metalloproteinases (MMP) by trophoblast cell lines after stimulation with *F. nucleatum* normalized to respective unstimulated controls **(A, B).** Data are presented as mean ± SEM. *p_adj_ < 0.05; **p_adj_ < 0.01; ***p_adj_ < 0.001 as analysed by Repeated Measures ANOVA with Dunnett’s multiple comparison post test, comparing each treatment against the corresponding control. Experiments were performed 5 (IL-8, MMP-2 and IL-6 in HTR8/SVneo) or 6 (CXCL1, MMP-9 and IL-6 in BeWo) times in duplicate. Each point represents the mean value of the replicates for each experiment. Ctl: control; Fus: ratio of *F. nucleatum* to cell number (if no number given ratio is 1); E. coli 1: ratio of *E.coli* to cell number = 1.

CXCL1, IL-8 and MMP-9 were only detectable in the supernatants of HTR8/SVneo, but not in BeWo nor JEG-3 supernatants (**Figure 4A**). The chemokine CXCL1 was induced after 24 h and 48 h of treatment with *F. nucleatum* at a ratio of 1 bacterium per HTR8/SVneo cell. Similarly, after 24 h an induction of IL-8 and MMP-9 secretion could be detected at a ratio of 1 bacterium per HTR8/SVneo cell. In contrast, *E. coli* stimulation induced the secretion of CXCL1, IL-8 and MMP-9 in al time points analyzed.

IL-6 and MMP-2 were detectable in the supernatants of both HTR8/SVneo and BeWo (**Figure 4B**).

The secretion of IL-6 by HTR8/SVneo was increased by *F. nucleatum* as well as *E. coli* stimulation in all time points. In contrast, the treatment of BeWo cells with *F. nucleatum* led to a decreased IL-6 secretion, while no effect of *E. coli* treatment could be observed. Similarly, *F. nucleatum* stimulation induced MMP-2 secretion from HTR8/SVneo, but decreased it in BeWo cells. No significant effect was observed after treatment with *E. coli* in both cell lines.

IL-1β concentration was below the detection threshold of 250 pg/mL in all trophoblast cell supernatants.

Similar to the previous results, HTR8/SVneo showed a stronger reaction as compared to BeWo. High bacterial concentrations led to a stronger secretory response in HTR8/SVneo (CXCL1, IL-6, IL-8, MMP-2 & -9). However, in BeWo cells responded with a decreased release of the investigated factors (IL-6, MMP-2) even with the low bacterial concentration.

### NF-κB Mediates TLR4 Dependent *F. nucleatum* Actions on HTR8/SVneo Cells

The differences in the response to bacteria between HTR8/SVneo and both, JEG-3 and BeWo cell lines, suggested that there may be differences in the ability to sense *F. nucleatum*.

**Figure 5:**
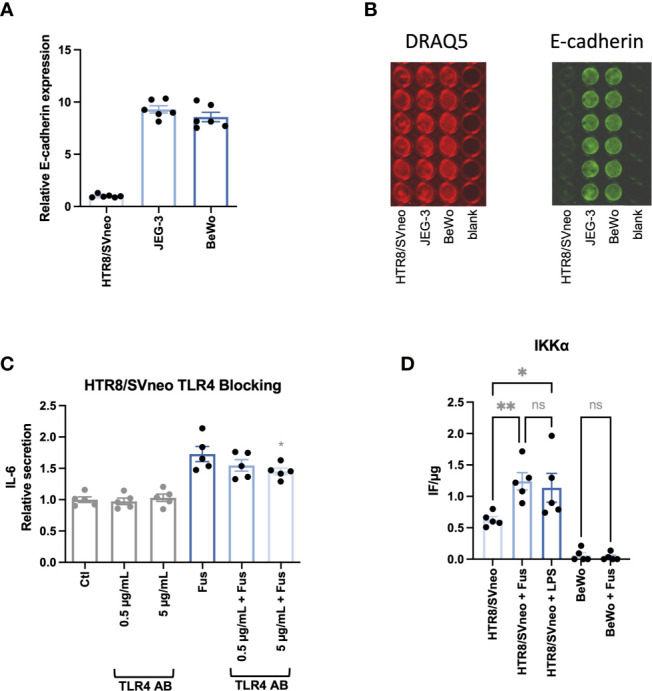
BeWo and JEG-3 cells, but not HTR8/SVneo cells express high levels of E-cadherin. IL-6 secretion in response to bacterial stimulation of HTR8/SVneo is partially TLR4 dependent. Bar graphs show E-cadherin expression in trophoblast cell lines normalized to HTR8/SVneo **(A)**. E-cadherin expression was normalized to cell number detected by cell nuclei staining with DRAQ5. Illustrative image of fluorescence signals of DRAQ5 binding and E-cadherin In-Cell Western analysis **(B)**. IL-6 secretion was assessed in HTR8/SVneo after stimulation with *F. nucleatum* in the presence or absence of a TLR4-blocking antibody **(C)**. The presence of the activated form of IKKα on HTR8/SVneo and BeWo cells was assessed after stimulation with *F*. *nucleatum* or LPS **(D)**. Data are presented as mean ± SEM. The experiment was performed once in sextuplicate **(A)**, six times in triplicate **(C)** or five times in duplicate **(D)**. *p_adj_ < 0.05; **p_adj_ < 0.01; ns, not significant, as analysed by Repeated Measures ANOVA with Dunnett’s **(C)** or Šidák’s **(D)** multiple comparison post test. Data comparison in **(C)** was performed on *F*. *nucleatum* treated cells employing the group without TLR4-blocking antibody as control (“Fus” column).

Since the interaction between *F. nucleatum* protein FadA and epithelial cells results from the interaction with E-cadherin (44), the basal expression of E-cadherin on the cell lines was assessed (**Figures 5A, B**). The relative E-cadherin signal (normalized as a ratio to HTR8/SVneo signal) was ~10 times higher in BeWo and JEG-3 than in HTR8/SVneo.

Besides the interaction with E-cadherin, gram-negative bacteria can be sensed by their LPS *via* TRL4 signalling and cause a pro-inflammatory reaction as observed in HTR8/SVneo. Interestingly, it has been observed that BeWo respond less sensitively to LPS stimulation than other trophoblast cells lines as JEG-3 and do not follow classical NF-κB pathway activation (77). In order to determine the impact of TLR4-dependent signalling, we performed the experiments in the presence and absence of a TLR4-blocking antibody (**Figure 5C**). The presence of the antibody led to a significant dose-dependent reduction of *F. nucleatum*-induced IL-6 secretion in HTR8/SVneo. Furthermore, *F. nucleatum* induced the activation of the NF-κB pathway, leading to increased phosphorylation of the The IκB kinase α (IKKα) in HTR8/SVneo while no activation of IKKα was detected in BeWo cells (**Figure 5D**).

**Figure 6:**
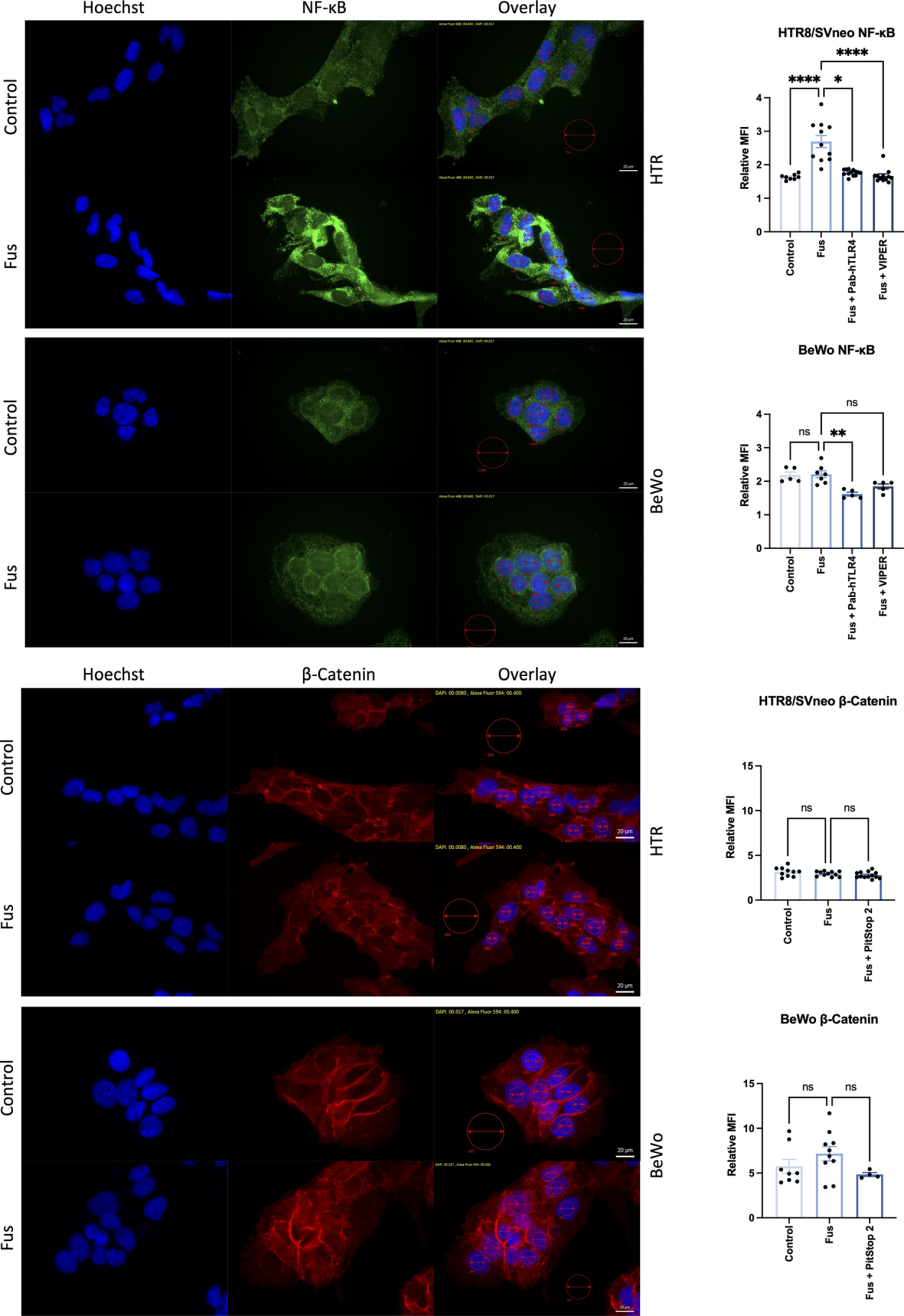
Inactivated *F. nucleatum* induces NF-κB and β-catenin nuclear translocation. Immunofluorescence of NF-κB (top; green) and β-catenin (bottom; red) of untreated or inactivated *F. nucleatum*-treated (1 h, MOI = 1) HTR8/SVneo and BeWo cells. Some wells were previously treated with a neutralizing antibody against TLR4 (PAb-hTLR4 (5 µg/mL), the viral inhibitory peptide of TLR4 (VIPER; 5 µM) or Pitstop 2 (known to interfere with E-cadherin/β-catenin signaling) 1 h before bacteria treatment. Nuclei were stained with Hoechst 33258 (blue). Pictures were taken at 60× and the mean fluorescence intensity (MFI) of each channel were quantified in the nuclei (small red circles). All pictures were taken using the same exposure time (green channel: 840 ms; red channel: 400 ms; blue channel: 17 ms). Data (left) depict the MFI (mean ± SEM) of either NF-κB or β-catenin normalized to background (big red circle) for each picture shown. Data comparison was performed by ANOVA Kruskall-Wallis test with Dunns multiple comparison test using *F. nucleatum* treated cells as control (“Fus” column). *p_adj_ < 0.05; **p_adj_ < 0.01; ****p_adj_ < 0.0001; ns, not significant.

To gain further insights into the signaling pathways triggered by *F. nucleatum* following TLR4 and E-cadherin activation, NF-κB and β-catenin were analyzed microscopically in the presence of inactivated *F. nucleatum* and inhibitors of TLR4 and E-cadherin pathways. Untreated HTR8/SVneo and BeWo cells showed cytoplasmic expression of NF-κB. After 1 h treatment, NF-κB was detected predominately close to and within the nucleus of HTR8/SVneo cells (**Figure 6**, top). The addition of TLR4-blocking antibody or the inhibitor TLR4-VIPER prior to bacterial treatment reverted this activation.

The transcription factor β-catenin mediates E-cadherin signals triggered by the binding of the *F. nucleatum* FadA adhesin molecule. BeWo cells displayed higher levels of β-catenin expression than HTR8/SVneo cells. Nuclear localization of β-catenin was found in a low number of cells BeWo, slightly more frequently after treatment with *F. nucleatum*. The use of the β-catenin inhibitor Pitstop 2 led to a slightly less, but not significant reduction of β-catenin signal after *F. nucleatum* treatment.

This data confirms that *F. nucleatum* triggers TLR4/NF-κB pathway activation and suggests that E-cadherin/β-catenin pathway is likely more predominant in BeWo than in HTR8/SVneo cells.

## Discussion

Although several studies support the idea that bacterial communities are present in the upper reproductive tract, their physiological impact remains still speculative. In this work, we have tested the hypothesis that the presence of low amounts of *F. nucleatum* can modulate trophoblast function without eliciting a major destructive inflammatory response.

It has been postulated that bacteria may exert a modulatory effect on trophoblast function through interactions between bacterial LPS and TLR4 expressed on the cell surface (36, 78). Both *E. coli* and *F. nucleatum* are gram-negative bacteria, thus they can induce LPS-mediated responses. Indeed, several studies addressed LPS-mediated effects of *F. nucleatum* in tumorigenesis and placental pathology (79–83). It is likely that the induction of pro-inflammatory responses we observed were LPS-mediated as well. However, certain responses differed between the treatments with *F. nucleatum* and *E. coli* (release of cytokines including chemokines).

As comparable amounts of bacteria have been used, discrepancies between both responses may be caused by other bacterial components than LPS. *F. nucleatum* has several virulence factors and is known to possess immunomodulatory properties, including a number of cell-surface components called adhesins (45, 49–51, 84). The adhesin FadA, for example, binds E-cadherin and activates NF-κB downstream (44). In the context of colorectal cancer, *F. nucleatum* is associated with the promotion of tumorigenesis and the modulation of the tumoral immune environment (44, 85, 86). At the same time, *F. nucleatum* has the ability to induce modifications of the extracellular matrix and promote tumor invasion (39, 41, 42, 58). In the fetomaternal interface, these processes are part of physiological adaptations that permit trophoblast invasion of uterine spiral arteries. Trophoblasts undergo phenotypical changes during placentation and in the course of pregnancy. This includes adaptations in changes of the expression of TLR4 and E-cadherin influencing presumably interactions with LPS and FadA, on the surface of *F. nucleatum*.

In our experiments, trophoblast cell lines responded differently to the same bacterial stimulation. In terms of antigen recognition, BeWo responds poorly to LPS stimulation and lacks LPS-mediated activation of the NF-κB pathway (77). We observed that HTR8/SVneo responded to *F. nucleatum* stimulation in a more sensitive way than BeWo and JEG-3. In contrast to BeWo and JEG-3, HTR8/SVneo E-cadherin expression levels were lower. This supports the idea that *F. nucleatum* shapes the responses of JEG-3 and BeWo by FadA-E-cadherin interaction. JEG-3 cells, which express both functional TLR4 and high E-cadherin levels, showed a mild or an intermediate reaction to bacterial stimulation. Cytokines in the supernatant of bacteria-treated JEG-3 were under the limit of detection.

The use of trophoblast cell lines with different TLR4 function and E-cadherin expression allowed us to evaluate two scenarios, one in which TLR4-LPS interaction would predominate over E-cadherin-FadA interactions (HTR8/SVneo), and a second one where E-cadherin is highly express and TLR4 is less functional (BeWo) (77). We speculate that the differences observed in the interaction between *F. nucleatum* and HTR8/SVneo, JEG-3 and BeWo cells depend on the balance between the relative expression of E-cadherin and the induction of TLR4-mediated signals. A deeper analysis of the activation of the signalling pathway depicted that, similar to LPS, *F. nucleatum* induced activation of the IκB kinase α (IKK-α), a downstream mediator of TLR4 activation pathway. Concomitantly, the treatment led to a nuclear translocation of NF-κB. Furthermore, the use of a neutralizing antibody against TLR4 resulted in reduce cytokine production after treatment with *F. nucleatum*.

In the BeWo cell line, no activation of the TLR4 pathway could be detected by multiplex analysis. However, nuclear translocation of NF-κB could be observed microscopically after 1 h treatment. In BeWo, the elevated expression of E-cadherin and β-catenin suggests a higher involvement of the E-cadherin/β-catenin complex in the *F. nucleatum*-mediated effects on BeWo cells than in HTR8/SVneo cells. Further research is needed to determine precisely the molecular components involved in the interaction between *F*. *nucleatum* on BeWo.

Besides cell-line specific responses, we observed that presumably LPS-mediated actions (those observed in HTR8/SVneo and that were similar to the stimulation with *E. coli*) were only significant after reaching relatively high concentrations of bacteria. On the other hand, LPS-independent effects, as we observed in BeWo cells, were also evident with low concentrations of fusobacteria. *F. nucleatum* is a bacterium with proven placental tropism (87–90) and *F. nucleatum* infections have been associated with intra-amniotic infection and the induction of preterm birth (91–93). The involvement of *F. nucleatum* in early pregnancy disorders needs to be further investigated. First trimester infections are associated to placenta development problems (94–97). In the context of malaria, *Plasmodium*-infection affects the placental vascular development, as seen by a reduced transport capacity, syncytiotrophoblast knotting, thickening of the basal membrane, decreased trophoblast invasion and inflammatory disorders (disruption of the cytokine milieu and immune cell recruiting) (98). Our data suggests that uncontrolled infections with *F. nucleatum* in early pregnancy might impact placental development as well.

However, the presence of bacteria does not necessarily indicate an infection. It has been observed that trophoblasts can modulate the response of immune cells to LPS, leading to contradictory effects between low and high dose stimulations (99). This has been discussed as a possible mechanism to prevent excessive pro-inflammatory reactions leading to fetal damage. The benefit of weak LPS stimulation to restore fertility has been observed in animal models. Cows with purulent vaginal discharge treated with a low dose of LPS showed improved pregnancy rate as compared to treatment with high LPS concentrations (100, 101). Although, eutherian mammal placentation varies in their invasive and opposing nature between fetus and maternal tissue (humans: hemochorial, ruminants: synepitheliochorial), it is driven by mild immunological activation, which is limited as exuberant activation would cause rejection. The studies describing mechanisms suppressing excessive pro-inflammatory responses at the fetomaternal interface suggest that the presence of bacteria in low concentrations or bacterial products can be well tolerated. Furthermore, it has been speculated that a weak, non-destructive activation of immune cells may actually be favorable in early pregnancy events as well (36, 37).

In order to evaluate possible mechanisms in which low, non-infective concentrations of bacteria may promote early pregnancy events, we studied the *F. nucleatum*-trophoblast interactions *in vitro*. In our experimental setup, we evaluated the role of increasing concentrations of *F. nucleatum* in a range which lies between 10 and 1 000 times lower than MOIs used in infection based *in vitro* experiments. Using this range, we aimed to detect the concentrations where the positive effects of *F. nucleatum* on trophoblast function overcome destructive excessive inflammatory responses. The analysis of the invasiveness of HTR8/SVneo depicts this concept perfectly, where a maximum effect can be observed around Fus0.1-1, while lower or higher concentrations seem to be less effective. Unfortunately, due to the fast migratory kinetics of HTR8/SVneo cells, it was not possible to perform the scratch assay at the same time point as the invasion assay. 12 h might be a precipitated time point to evidence positive effects of lower *F. nucleatum* concentrations on cell migration.

**Figure 7:**
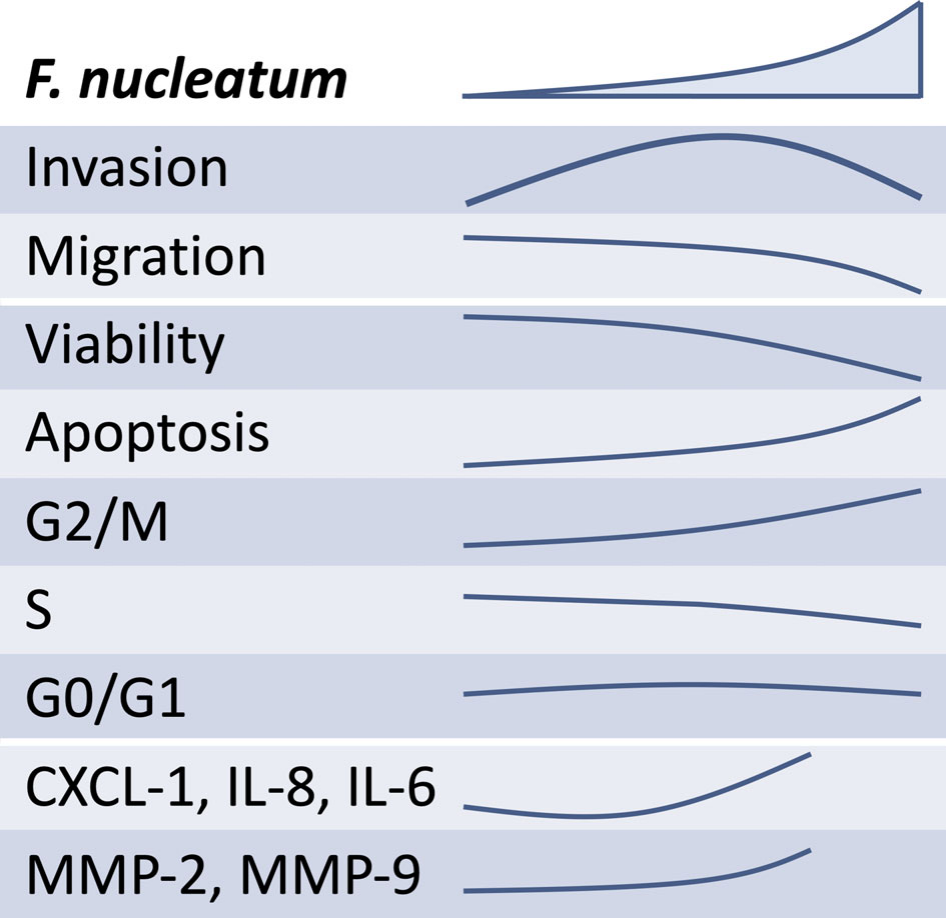
Overview of the schematic effects of rising inactivated *F. nucleatum* concentrations on HTR8/SVneo. Main results of HTR8/SVneo trophoblastic cells in response to *in vitro* stimulation with *F. nucleatum* are summarized. *F. nucleatum* induced HTR8/SVneo invasion, secretion of soluble mediators (CXCL1, IL-6 and IL-8) and metalloproteinases (MMP-2 and MMP-9). As concentrations of *F. nucleatum* increased, these did not improve invasiveness, hindered migration, reduced cell viability and induced alterations in the cell cycle.

It can be speculated that the lower the concentration of *F. nucleatum* is, the weaker its effect on the release of soluble mediators that promote trophoblast invasiveness shall be (see schematic overview, **Figure 7**). In contrast, as the concentration of *F. nucleatum* increases, the excessive inflammatory effects on trophoblast may negatively affect their function. Indeed, the highest *F. nucleatum* concentration significantly dampened trophoblast migration, which also brought trophoblast invasion down to control levels.

The analysis of cell survival and the apoptosis rate after *F. nucleatum* treatment suggests that the negative effects on migration observed might be related to the reduced viability or an altered cell cycle after treatment. These negative effects of *F. nucleatum* increased with the concentration and were more evident in the HTR8/SVneo cell line.

After evidencing the effects that might negatively impact on trophoblast function, we focused on the factors that may improve it, especially under treatment with low concentrations of *F. nucleatum*. A factor by which bacteria could promote placentation is by induction of MMPs which facilitate trophoblast invasion. MMPs dysregulation is associated to pregnancy problems (102). Deficient MMP expression may lead to hypertensive disorder and preeclampsia. Excessive MMP release, however, can lead to dysfunctional placentation. In this concern, we observed that *F. nucleatum* could modulate MMP secretion.

We have also explored the capacity of bacteria to affect the release of immune mediators that may affect directly or indirectly functional aspects of trophoblast biology. Trophoblasts release immune mediators that: 1) recruit and modulate the function of several leukocytes populations (decidual NK cells, macrophages, etc) and 2) collaborate with crucial steps of placentation (103, 104). As the treatment with *F. nucleatum* affected some of these cytokines, we speculate that these may later influence leukocyte recruitment and function and indirectly trophoblast function. In this scenario, chemokines induced by *F. nucleatum* may act synergistically with the arrival of leukocytes that are known to be important players of placental development, as macrophages and NK cells.

The fact that the cytokine secretion in HTR8/SVneo was induced both in response to *F. nucleatum* and *E. coli* treatment led us to a hypothesis that this effect was mediated by LPS. Furthermore, there was no induction of cytokine secretion by BeWo cells, which have a less sensitive TLR4-pathway. Finally, we showed that blocking or inhibition of TLR4 reduced the NF-κB activation and cytokine secretion in *F. nucleatum*-treated HTR8/SVneo cells. We postulated that these interactions might be subjected to spatiotemporal conditions in the course of pregnancy, since trophoblast undergoes local and temporal changes in the expression of both TLR4 and E-cadherin. During first trimester, TLR4 is expressed by villous cytotrophoblast (CTB) and extravillous trophoblast cells (EVT), but not by syncytiotrophoblasts (105, 106). At term, TLR4 is expressed predominantly by syncytiotrophoblasts (105, 107). This pattern is thought to protect the first trimester fetus from deleterious pro-inflammatory responses caused by bacteria. On the other hand, E-cadherin is expressed in CTB but it is downregulated as EVTs acquire a more invasive phenotype.

In this scenario, *F. nucleatum* might interact with EVT secreting MMPs and inducing invasion through the decidual extracellular matrix, pro-inflammatory cytokines (including chemokines) to recruit and interact with decidual leukocytes. The presence of low concentrations of *F. nucleatum* could support the function of EVT. CTB, on the contrary, are in closer contact to the growing fetus. An excessive pro-inflammatory environment generated by activation of CTB could threaten fetal health.

The presence of bacteria in the placenta has been reported by histological techniques and later further investigated by molecular-based methods (29, 108, 109). Furthermore, as these studies are based on the detection of DNA, it cannot be clearly distinguished between bacteria and their products. In our experiments, however, we used inactivated cells. This means that bacterial components that reach target cells may induce similar responses. Furthermore, several gram-negative bacteria including *F. nucleatum* are characterized by the production and release of outer membrane vesicles (OMV). OMV play different roles (including bacterial communication, the modulation of virulence and immune response). As they are small enough to penetrate mucosal barriers, a remote modulatory mechanism of trophoblast function by *F. nucleatum* cannot be ruled out.

Based on our data, we suggest that the presence of low-concentration of commensal bacteria or bacterial products do not represent a threat to early pregnancy *per se*. Although the used concentrations only approached *in vivo* amounts, low bacterial concentrations may mildly stimulate trophoblast cells and support their invasive character. As the upper reproductive tract microbiome may deliver clues to possible, but yet unknown physiological regulation of trophoblast function, we encourage further research to elucidate their constructive role during early pregnancy. Precisely during the review process of this manuscript, a new study showing that *Lactobacillus crispatus* can promote HTR-8/SVneo invasion supports this idea and reinforces the need for deeper research on this field (110).

## Data Availability Statement

The raw data supporting the conclusions of this article will be made available by the authors, without undue reservation.

## Author Contributions

MH and RE performed experiments, analysed data, and contributed to the elaboration of the manuscript. JE performed experiments. MZ contributed with reagents, the design of experiments, and the writing of the manuscript. DM conceived and designed the experiments, analysed data, wrote the paper, and supervised the work. All authors contributed to the article and approved the submitted version.

## Funding

This study was supported by intramural funding from Greifswald University. We also acknowledge the support of the Research Network Molecular Medicine (Forschungsverbund Molekulare Medizin, FVMM, FOVB-2021-10).

## Conflict of Interest

The authors declare that the research was conducted in the absence of any commercial or financial relationships that could be construed as a potential conflict of interest.

## Publisher’s Note

All claims expressed in this article are solely those of the authors and do not necessarily represent those of their affiliated organizations, or those of the publisher, the editors and the reviewers. Any product that may be evaluated in this article, or claim that may be made by its manufacturer, is not guaranteed or endorsed by the publisher.
